# Multimodality Molecular Imaging-Guided Tumor Border Delineation and Photothermal Therapy Analysis Based on Graphene Oxide-Conjugated Gold Nanoparticles Chelated with Gd

**DOI:** 10.1155/2018/9321862

**Published:** 2018-05-07

**Authors:** Xibo Ma, Yushen Jin, Yi Wang, Shuai Zhang, Dong Peng, Xin Yang, Shoushui Wei, Wei Chai, Xuejun Li, Jie Tian

**Affiliations:** ^1^CAS Key Laboratory of Molecular Imaging, Institute of Automation, Chinese Academy of Sciences, Beijing 100190, China; ^2^Department of Orthopedics, Chinese PLA General Hospital, Beijing 100853, China; ^3^Institute of Biomedical Engineering, School of Control Science and Engineering, Shandong University, Jinan, Shandong 250061, China; ^4^Department of Neurosurgery, Xiangya Hospital, Central South University, Changsha 410008, China

## Abstract

Tumor cell complete extinction is a crucial measure to evaluate antitumor efficacy. The difficulties in defining tumor margins and finding satellite metastases are the reason for tumor recurrence. A synergistic method based on multimodality molecular imaging needs to be developed so as to achieve the complete extinction of the tumor cells. In this study, graphene oxide conjugated with gold nanostars and chelated with Gd through 1,4,7,10-tetraazacyclododecane-N,N′,N,N′-tetraacetic acid (DOTA) (GO-AuNS-DOTA-Gd) were prepared to target HCC-LM3-fLuc cells and used for therapy. For subcutaneous tumor, multimodality molecular imaging including photoacoustic imaging (PAI) and magnetic resonance imaging (MRI) and the related processing techniques were used to monitor the pharmacokinetics process of GO-AuNS-DOTA-Gd in order to determine the optimal time for treatment. For orthotopic tumor, MRI was used to delineate the tumor location and margin* in vivo* before treatment. Then handheld photoacoustic imaging system was used to determine the tumor location during the surgery and guided the photothermal therapy. The experiment result based on orthotopic tumor demonstrated that this synergistic method could effectively reduce tumor residual and satellite metastases by 85.71% compared with the routine photothermal method without handheld PAI guidance. These results indicate that this multimodality molecular imaging-guided photothermal therapy method is promising with a good prospect in clinical application.

## 1. Introduction

Hepatocellular carcinoma (HCC) is the second most common cancer for death rate in the world and claims more than 700000 lives per year worldwide [1]. With advances in surgical techniques (resection, transplantation, and thermal ablation) and imaging techniques, 30% of patients are diagnosed and cured at early stage [2]. However, the management of 70% of patients with more-advanced stages remains challenging [3]. Besides surgical method, photothermal therapy has garnered the attention because of its tumor destruction ability [4]. Photothermal therapy utilizes photosensitizers to convert light to heat, which could destroy the tumor tissues. Cancer cells take up the photosensitizers, resulting in cell death because of photoablation. To avoid nonspecific heating of healthy cells, photosensitizers must be selectively taken up by cancer cells.

Therefore, designing a high absorption photosensitizer is essential to generate as much heat as possible from optical energy for photothermal therapy. In recent years, graphene oxide (GO) garnered the attention as a promising material for biomedical applications as a photoabsorber for photothermal therapy and photoacoustic imaging (PAI) due to its superior optical absorption in the NIR and high photothermal conversion efficiency and nanocarrier feature through the *π*-*π* stacking between drugs and GO nanosheets [5–7]. Functionalized GO is widely used as an efficient theranostic agent due to the existence of sp2 domain with hydroxyl, epoxy, and carboxyl groups [8]. We previously reported the ability of PLA microcapsules containing GO and Au nanoparticles as a theranostic system for multimodal imaging-guided cancer therapy [9, 10]. However, the micron scale agent cannot easily accumulate at the tumor site through the blood circulation system.

Additionally, the tumor margin must be delineated more accurately so that the treatment can be carried out more precisely to avoid damaging normal tissues. Simultaneously, the photothermal materials should accumulate to its peak value at the tumor site so that the irradiation could be initiated. Therefore, developing an effective imaging technique and analysis method is imperative to detect the tumor margin and monitor the pharmacokinetics process. Molecular imaging, especially optical molecular imaging, has been recently used to monitor the antitumor efficacy and calculate the location of orthotopic tumors [11–13]. However, single-modality imaging technique has its limitations in tumor margin delineation and precise photothermal therapy. Multimodality molecular imaging including MRI, PAI, and optical imaging combined with image processing technologies could solve the above problems.

In this study, GO-AuNS-DOTA nanoparticles chelated with Gd (GO-AuNS-DOTA-Gd) were prepared and used in photothermal therapy against HCC. MRI was used to delineate the tumor margin* in vivo* before treatment; PAI was used to monitor the pharmacokinetics process of GO-AuNS-DOTA-Gd and real-time detect the tumor location during treatment, and bioluminescent imaging was used to evaluate the antitumor therapy. Compared with the current photothermal methods, this synergistic method based on this synergistic method could improve treatment efficacy by 36.36% evaluated by BLI for subcutaneous tumor. For orthotopic tumor, this method could effectively reduce tumor residual and satellite metastases by 85.71% compared with the routine photothermal method without handheld PAI guidance.

## 2. Materials and Methods

### 2.1. Materials

Hydrogen tetrachloroaurate (III) hydrate (HAuCl_4_·4H_2_O), sodium citrate, and sodium borohydride (NaBH_4_) were obtained from Sinopharm Chemical Reagent Co., Ltd (Shanghai, China). Deionized water (DI water, 18.2 MΩ/cm^2^) from the Milli-Q gradient system was used in all experiments. CellTiter96® AQ_UEOUS_ One Solution Cell Proliferation Assay (MTS, batch No. G358A) was purchased from Promega Company (Madison, WI, USA).

### 2.2. Preparation of GO-AuNS-DOTA Nanoparticles Chelated with Gd

The preparation of* GO-AuNS-DOTA nanoparticles chelated with Gd* was carried out using the method reported in [14, 15]. Briefly, 5 *μ*g/mL graphene oxide was mixed with 1200 *μ*L of 0.1 M HEPES and then 2.4 *μ*L of HAuCl_4_ (0.1 M) was added to the mixture. Subsequently, GO-AuNS hybrids were centrifuged at 5000 rpm for 10 min to remove HEPES and any free gold nanostars in the supernatant. The precipitate was then resuspended in water.

To prepare the GO-AuNS-DOTA nanoparticles, GSH (5.0 mg) was added to the above solution with stirring overnight to form the nanoconstruct (GO-AuNS-GSH). After 12 h, the solution was centrifuged at 5000 rpm for 10 min. The precipitate was retained and DI water (1.0 mL) was added to redissolve the precipitate. Meanwhile, DOTA-NHS was dissolved into DI water under stirring at 55°C, followed by addition of GdCl_3_·6H_2_O in 5 mL water. The molar ratio of Gd to DOTA-NHS was 3 : 1; after chelating for 30 min, the GO-AuS-GSH was added to this solution and stirred for 24 h at room temperature to form the GO-AuNS-DOTA nanoparticles chelated with Gd (GO-AuNS-DOTA-Gd).

### 2.3. Characterization of GO-AuNS-DOTA Nanoparticles

The size distribution and zeta potential of GO-AuNS-DOTA nanoparticles were measured using a ZetaPlus dynamic light scattering instrument (Brookhaven Instruments) after dispersion of the nanoparticles at an appropriate concentration (200 *μ*g/mL). Six replicate samples of each type of vesicles were analyzed at a scatter angle of *θ* = 90° and a wavelength of 660 nm at room temperature.

The morphology of GO-AuNS-DOTA nanoparticles was observed by transmission electron microscopy (TEM, Tecnai™ G2 at 200 kV). Copper grids with carbon coating were used for sample preparation. First, nanoparticles were dispersed in deionized (DI) water at 0.2 mg/mL concentration. Then, 10 *μ*L of nanoparticle suspension was placed on the grid and absorbed for 30 seconds. Finally, the grid was washed with DI water and dried at room temperature in a draught cupboard.

The UV-Vis-NIR absorption spectra of the GO-AuNS-DOTA nanoparticles were acquired with a UV-Vis spectrometer (Varian 4000, USA). The amount of Gd and Au in the nanoparticles was quantified by the inductively coupled plasma (ICP) analysis.

### 2.4. In Vitro Photothermal Heating Experiments

To examine the temperature elevation efficiency, different concentrations of GO-AuNS-DOTA nanoparticles dispersed in DI water were under the irradiation of an 808 nm laser with an output power of 2 W. The temperature of all samples was monitored and periodically confirmed by a digital thermometer with a thermocouple probe every 10 seconds. DI water was irradiated as a control. The heating curve was determined by plotting the measured temperatures.

To confirm the photothermal stability of GO-AuNS-DOTA nanoparticles, solutions of 50 *μ*g/mL GO-AuNS-DOTA nanoparticles were irradiated with an 808 nm NIR laser for 10 min (LASER ON), followed by natural cooling to room temperature without NIR laser irradiation for 30 min (LASER OFF). This cycle was repeated five times and the heating curve of the five repeated cycles was plotted to characterize the photothermal stability of nanoparticles.

### 2.5. In Vitro Photothermal Effect against Cancer Cells

#### 2.5.1. Cell Culture

Firefly luciferase- (fLuc-) labeled human HCC (HCC-LM3-fLuc) cells were cultured in Dulbecco's Modified Eagle's Medium (DMEM) supplemented with 10% fetal bovine serum (FBS), 100 *μ*g/mL penicillin, and 100 *μ*g/mL streptomycin in a humidified incubator at 37°C in a 5% CO_2_ atmosphere. The cells were passaged every 3-4 days.

#### 2.5.2. Photothermal Effect of the Nanoparticles against HCC-LM3-fFluc Cells

Photothermal cytotoxicity of GO-AuNS-DOTA was evaluated on HCC-LM3-fFluc cells. For qualitative analysis, HCC-LM3-fFluc cells were seeded onto 6-well plate at a density of 2 × 10^5^ cells per well and incubated at 37°C in a humidified atmosphere containing 5% CO_2_ overnight. After incubation with different concentrations of GO-AuNS-DOTA nanoparticles for 4 h, the medium containing nanoparticles was replaced with fresh medium, and the cells were exposed to an 808 nm NIR laser with an output power of 2 W for 5 min. After incubation for 2 h, the cells were stained with propidium iodide (PI) to evaluate the photohyperthermic effect of the nanoparticles on cancer cells and the fluorescence images were collected with an inverted fluorescence microscope (Leica, Germany) equipped with a cooled CCD camera.

For quantitative evaluation of the phototoxicity, HCC-LM3-fFLuc (1 × 10^4^ cells per well) were incubated in 96-well plate and incubated overnight to allow the cells to attach to the surface of the wells. After incubation with GO-AuNS-DOTA for 4 h, the medium containing nanoparticles was replaced with fresh medium and the cells were irradiated with a diode NIR laser centered at 808 nm at an output power of 6 W/cm^2^ for different times. After incubation for 4 h, cell viability was determined by Trypan Blue assay in quadruplicate.

#### 2.5.3. Tumor Models

Balb/c mice were obtained from the Department of Experimental Animals, Peking University Health Science Center, and experiments were performed under protocols approved by the Institutional Animal Care and Use Committee at Peking University. The HCC-LM3-fFLuc subcutaneous tumor-bearing mice were established by injecting 6 × 10^6^ cells into the right flanks and used for photothermal therapy when the tumor volume reached about 160 mm^3^.

### 2.6. In Vivo Target Study of GO-AuNS-DOTA-Gd and Photothermal Therapy Based on Subcutaneous Tumor Model and Multimodality Molecular Imaging

#### 2.6.1. In Vivo Photoacoustic Imaging

HCC tumor-bearing mice were injected with GO-AuNS-DOTA-Gd via the tail vein (80 *μ*L per mouse). The probe concentration in the blood circulation and tumor was monitored by PAI. The PAI was acquired by a multispectral photoacoustic tomography system (inVision 128, iTheraMedical GmbH, Munich, Germany). Five excitation wavelengths, 715 nm, 730 nm, 760 nm, 800 nm, and 850 nm, were applied for* in vivo* PAI in order to resolve the equation of drug distribution. The tumors were imaged at preinjection, 1, 2, 3, 5, 7, 12, and 24 h after injection of the probe. For tumor imaging, the mice were scanned from the neck to stomach with a step distance of 0.3 mm. Multispectral processing was used to unmix the signal of the target probe.

#### 2.6.2. In Vivo MRI and Tumor Margin Delineation and Three-Dimensional Image Processing

*In vivo* MRI was acquired at preinjection, 2, 5, 7, and 12 h after the tail injection of the probe by an M3™ Compact High-Performance MRI System (Aspect Imaging, Industrial Area Hevel Modi'in, Israel) using 23 inner diameter solenoid coil (named Mouse Head L25 D23). The scanning parameters were set as follows: modality *T*_1_, slice thickness: 0.8, slice gap: 0.1, TR: 6000. Simultaneously, we determined the longitudinal relaxivity of GO-AuNS-DOTA-Gd NPs under a 3.0-T MRI scanner.

After image acquisition, tumors and outline in mice were segmented according to the gray value difference and the segmented regions of interest (ROI) were visualized using 3D slicer [16]. The maximal radium was deemed to half of maximum value of the distance between two random voxels at the tumor surface, as shown in the following formula:

(1) $$\begin{array}{c} R_{max}=\frac{1}{2}\underset{i,j=1:N}{\max}\sqrt{{\left(x_{i}-x_{j}\right)}^{2}+{\left({y_{i}-y}_{j}\right)}^{2}+{\left(z_{i}-z_{j}\right)}^{2}}, \end{array}$$

where (*x*_*i*_, *y*_*i*_,*z*_*i*_), (*x*_*j*_, *y*_*j*_, *z*_*j*_) are two random points on the surface of the segmented tumor.

The average MRI gray value of the tumor in 18 slices was calculated using Philips DICOM Viewer R3.0 SP3 [17]. The tumor volume was calculated according to the following formula:

(2) $$\begin{array}{c} V=\overset{N}{\underset{i=1}{\sum }}v_{0}n_{i}, \end{array}$$

where *n*_*i*_ is the number of voxels of the tumor ROI in *i*th slice, *v*_0_ is the volume of every voxel, and *N* = 18 is the number of slices.

Meanwhile, the average gray value contrast ratio was used to demonstrate the discrimination ability of the probe for tumor and normal tissue, which was calculated using the following formula:

(3) $$\begin{array}{c} CR=\frac{\sum_{i-1}^{N}\left({AG}_{i}/{AR}_{i}\right)}{N}, \end{array}$$

where AG_*i*_ is the average gray value of the tumor ROI in the *i*th slice and AR_*i*_ is the average gray value of region around the tumor in the *i*th slice. Here, the region around the tumor was acquired through the following two steps. *N* = 18 is the number of slices. First, the tumor ROI was carried out morphological dilation until the area was twice the tumor ROI, except the background. Then, the total area minus the area of tumor ROI was considered the region around tumor.

#### 2.6.3. In Vivo Positron Emission Computer Tomography (PET) Using ^18^*F*-FDG

PET scans were acquired with a micro-PET GENISYS 4 scanner system (Sofie Biosciences, USA) after the mice received a tail-vein injection of ^18^F-FDG (100 *μ*Ci). The mouse was scanned 30 minutes after injection with a 10 min static PET scan. During the scan, the mouse was anesthetized using 2% isoflurane. The image was reconstructed using a 3-dimensional maximum a priori algorithm and into 9696208 matrices with an isometric voxel size of 0.46 mm^3^.

### 2.7. Photothermal Therapy

Female Balb/c mice bearing HCC-LM3-fLuc tumors were divided into 8 groups (7 mice per group) when the tumor volume reached about 150 mm^3^. Three groups were treated with 80 *μ*L of GO-AuNS (GO: 2 mg/mL, Au: 1.31 mg/mL) via tail veil injection and laser treatment was carried out at 2, 5, and 12 h after injection (named (D2) group, (D5) group, and (D12) group), respectively. One group was only treated with 80 *μ*L of GO-AuNS via tail vein but did not receive laser treatment. Two groups were treated with 80 *μ*L of GO-AuNS via intratumor injection and one received laser treatment, while the other group did not. Two groups were treated with 80 *μ*L of 0.9% saline via tail-vein injection and one received laser treatment, while the other group did not. For the laser treatment groups, the tumors were irradiated by an optical fiber coupled with an 808 nm NIR laser (Hi-Tech Optoelectronics Co., Ltd. Beijing, China) at the power density of 0.2 W/cm^2^ for 5 min. The temperature around the tumor after irradiation was collected by using an infrared camera.

### 2.8. Orthotopic Tumor Models and Multimodality Molecular Imaging-Guided Cancer Treatment

The 21 orthotopic tumor models were established by injecting 2 × 10^6^ HCC-LM3-fLuc tumor cells into the liver of each BALB/c nude mouse. The mice were randomly separated into 3 groups each consisting of 7 mice. One group (control) was injected i.v. with PBS. The other two groups (treatment) were injected i.v. with GO-AuNS-Gd on the 10th day.* In vivo* MRI was acquired at preinjection, 1, 2, 3 and 12 h after the tail injection. Photothermal therapy was conducted on all mice of the three groups. The process included the following two steps. First, the abdomen of mice was opened and the liver was exposed to the air. Then handheld photoacoustic imaging scanning was conducted on mice of one treatment group and, according to the PAI which indicated the tumor location, photothermal therapy was conducted using an optical fiber coupled 808 nm NIR laser at the power density of 0.2 W/cm^2^ for 6 min.

### 2.9. Evaluation of the Antitumor Efficacy

After treatment,* in vivo* BLI obtained through an IVIS Spectrum imaging system (PerkinElmer, USA) was used to evaluate the antitumor efficacy. For subcutaneous tumor models, the image was acquired at the 8th minute after the injection of 100 *μ*L D-Luciferin (15 mg/mL) into the abdomen of the experimental mice on days 0, 2, 5, 8, 11, 15, and 20 after treatment. For orthotopic tumor models, the image was acquired on 5th day. The antitumor efficacy was calculated according to the following:

(4) $$\begin{array}{c} \text{Antitumor efficacy}=\left(\;1-\frac{BI_{ex}}{BI_{control}}\;\right)\times 100\%, \end{array}$$

where BI_ex_ is the average bioluminescent intensity of mice tumors in experiment group. BI_control_ is the average bioluminescent intensity of mice tumors in control group.

### 2.10. Drug Toxicity Assessment and Biodistribution Study

For subcutaneous tumor experiments, to assess the toxicity of GO-AuNS-DOTA-Gd and photothermal efficacy, six organs including the heart, liver, spleen, lung, kidney, and tumor were harvested after euthanasia on the 21st day after tail injection and stained with hematoxylin and eosin (H&E). Mouse weight was also acquired on days 0, 2, 5, 8, 11, 15, and 20 after treatment and plotted as a weight graph in OriginPro for each group. For orthotopic tumor experiments, tumors from PBS group and probe group after treatment were harvested and stained with H&E.

For biodistribution assay, HCC-LM3-fLuc subcutaneous tumor-bearing mice were sacrificed at 2 h, 5 h, and 12 h after* i.v.* injection of GO-AuNS-DOTA-Gd (200 *μ*L per mice, *n* = 3). Major organs including heart, liver, spleen, lung, kidney, intestines, brain, and tumor were collected, weighed, and then solubilized by aqua regia. The concentrations of Au and Gd in those tissue lysate samples were measured by inductively coupled plasma mass spectrometry (ICP). The levels of GO-AuNS-DOTA-Gd in various organs are presented as the percentage of injected dose per gram of tissue (ID *μ*g/g). In addition, the concentrations of Au and Gd in liver tumor and normal liver including above tissues based on the orthotopic tumor model were measured by ICP. The mice were sacrificed at 1 h and 12 h after i.v. injection of GO-AuNS-DOTA-Gd (200 *μ*L per mice, *n* = 3).

### 2.11. Statistical Analysis

Quantitative data are expressed as mean ± SD. Results of two sets were compared using one-way analysis of variance (ANOVA) and a Student *t*-test, and *p* values < 0.05 were considered statistically significant difference.

## 3. Results

### 3.1. Preparation and Characterization of the GO-AuNS-DOTA Nanoparticles

**Figure 1:**
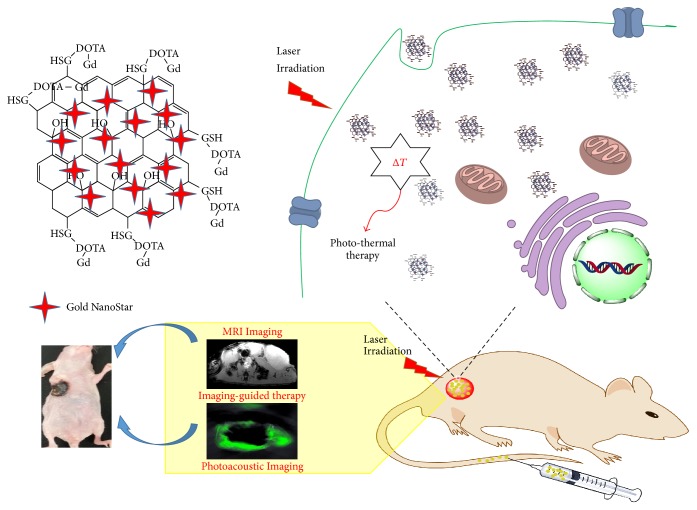
Synthesis of GO-AuNS-DOTA-Gd, imaging-guided tumor margin detection, and photothermal therapy analysis.

**Figure 2:**
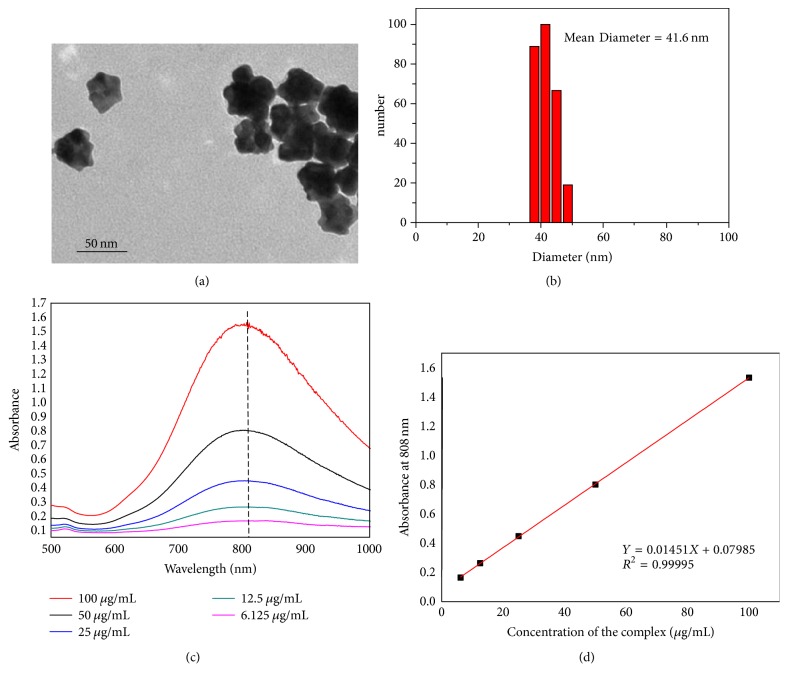
(a) TEM image of GO-AuNS-DOTA-Gd nanoparticles; (b) distribution of the nanoparticles analyzed by DLS; (c) increased UV-Vis-NIR absorption spectra of various concentrations of GO-AuNS-DOTA nanoparticles in RPMI-1640 culture medium; (d) the absorbance of GO-AuNS-DOTA-Gd nanoparticles dispersed in RPMI-1640 culture medium at 808 nm increased as the concentration increased.

The synthesis of GO-AuNS-DOTA-Gd nanoparticles was carried out according to Figure 1. The mean diameter of GO-AuNS-DOTA-Gd nanoparticles was 41.6 nm as determined by dynamic laser scattering (DLS) and TEM (Figures 2(a) and 2(b)) and zeta potentials of the nanoparticles in DI water and PBS (pH 7.4) were −17.2 mV and −2.3 mV, respectively. The amount of Gd and Au in the nanoparticles was 0.77 mg Gd and 1.31 mg Au per 2 mg GO according to (ICP) experiments, respectively.

GO-AuNS-DOTA showed a distinct absorption peak at 810 nm due to the existence of Au nanostars. UV-Vis-NIR spectra of GO-AuNS-DOTA in RPMI-1640 culture medium demonstrated (Figure 2(c)) no obvious difference in the absorption spectra and suggested good colloidal stability of GO-AuNS-DOTA nanoparticles. Moreover, absorption increased linearly as the nanoparticle concentration increased (Figure 2(d)), indicating the excellent dispersity of GO-AuNS-DOTA nanoparticles in culture medium.

### 3.2. In Vitro Photothermal Effects of GO-AuNS-DOTA Nanoparticles

**Figure 3:**
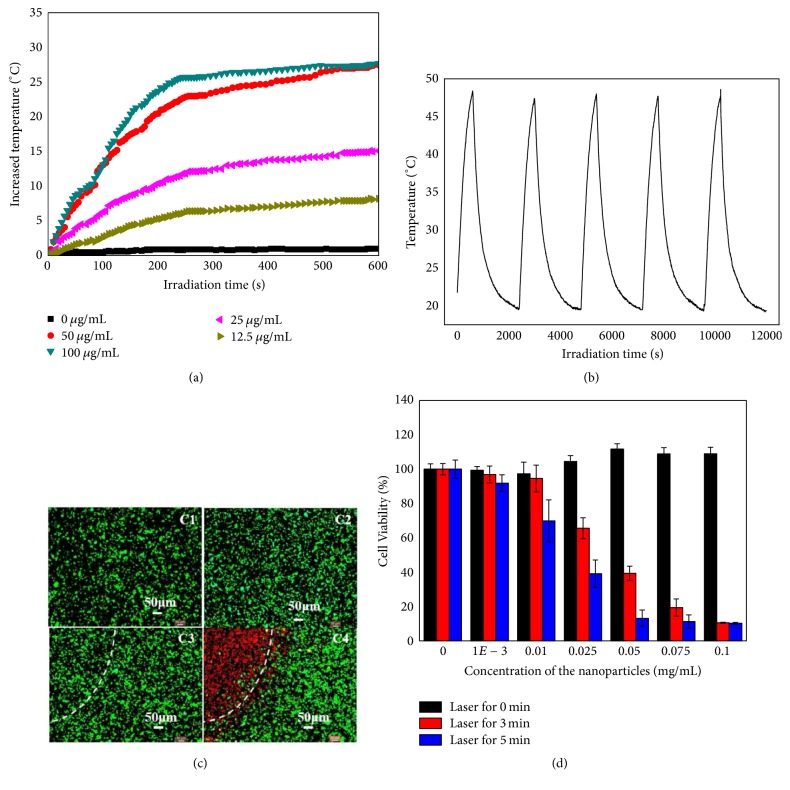
(a) Temperature elevation of aqueous solution of GO-AuNS-DOTA-Gd nanoparticles at different concentrations over 10 min under NIR laser irradiation (808 nm, 2 W) recorded every 10 seconds with a digital thermometer; (b) temperature elevation of aqueous solution of GO-AuNS-DOTA-Gd nanoparticles over five LASER ON/OFF cycles of NIR laser irradiation (808 nm, 2 W) (LASER ON time: 10 min; LASER OFF time: 30 min); (c) photothermal destruction of HCC-LM3-fFluc cells with different treatments. C1: no irradiation and no agent; C2: agent of 25 *μ*g/mL only; C3: 5 min irradiation, no agent; C4: 5 min irradiation, agent of 25 *μ*g/mL. White dashes indicate the laser boundary; dead cells are labeled in red, while viable cells are labeled in green; (d) cell viability after treatment with different concentrations of GO-AuNS-DOTA-Gd nanoparticles and different NIR laser irradiation time. ^*∗∗*^*p* < 0.01 and ^*∗*^*p* < 0.05.

The temperature of nanoparticle solutions showed a rapid increase within 4 min of laser irradiation, and the temperature elevation rose as the nanoparticle concentration increased (Figure 3(a)). In contrast, no apparent temperature change was detected for PBS. As shown in Figure 2(b), the temperature increased 27°C after the first LASER ON and, in the next four cycles, no significant variation of temperature elevation was observed.

The result of* in vitro* photothermal experiment indicated that cancer cells in three control groups, including GO-AuNS-DOTA group, laser irradiation group, and group without any treatments, maintained their healthy state (Figure 3(c)). In contrast, cells from experimental groups with both GO-AuNS-DOTA and laser irradiation treatment showed a bright red fluorescence in the laser irradiation region, indicating that GO-AuNS-DOTA could mediate the photothermal destruction of HCC-LM3-fFluc cells.

Cell viability using MTT method (Figure 3(d)) demonstrated GO-AuNS-DOTA nanoparticles without laser irradiation exhibited little cytotoxicity on HCC-LM3-fFluc cells. In contrast, GO-AuNS-DOTA nanoparticles followed by laser irradiation (808 nm and 2 W/cm^2^) induced cell cytotoxicity in a dose- and time-dependent fashion.

### 3.3. In Vivo Target Study of GO-AuNS-DOTA-Gd Based on Subcutaneous Tumor Model and Multimodality Molecular Imaging

**Figure 4:**
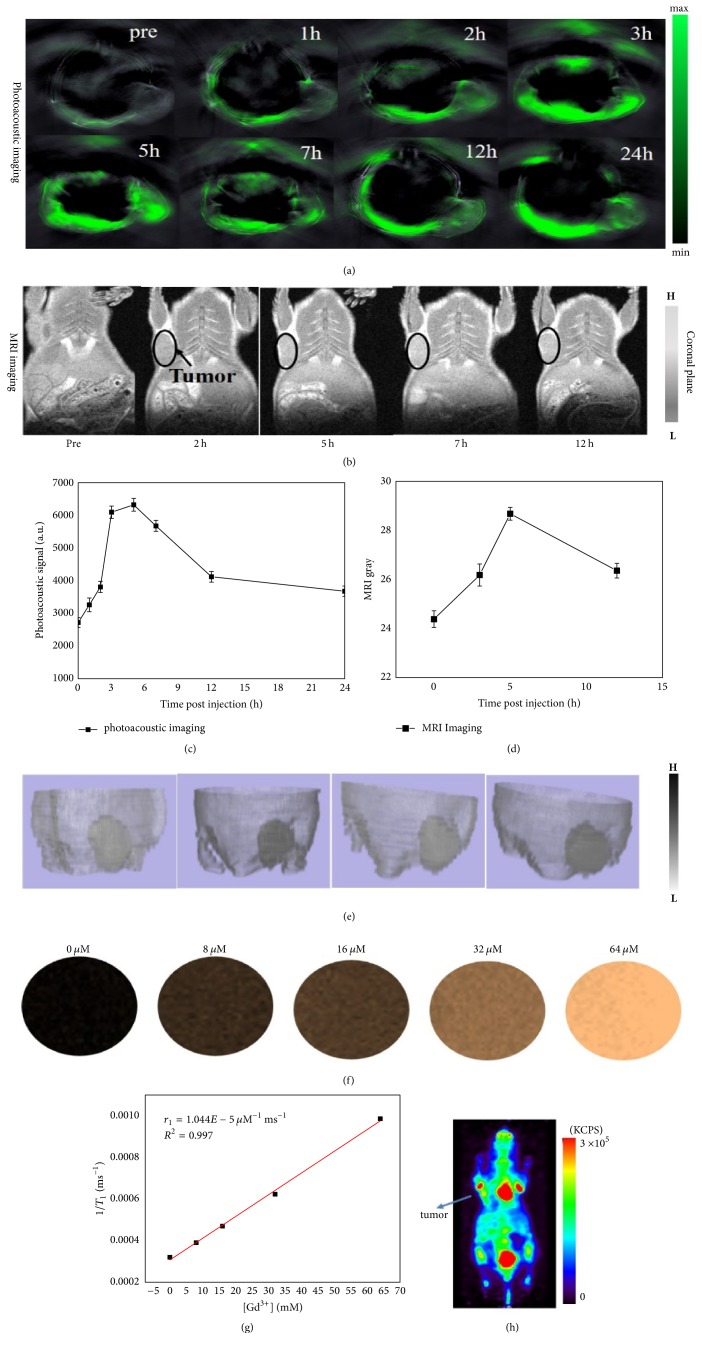
(a) Photoacoustic signal distribution of GO-AuNS-DOTA-Gd in tumor regions at different time points before and after injection; (b) MRI image of GO-AuNS-DOTA-Gd on the coronal plane in tumor regions at different time points before and after injection; (c) quantitative analysis of the PAI signal in the tumor regions; (d) quantitative analysis of MRI gray intensity in the tumor regions; (e) reconstructed 3D image and 3D visualization of tumor and its surrounding tissue based on the MRI slice images; (f) MRI image of GO-AuNS-DOTA-Gd at different concentrations; (g) linear relationship between *T*_1_ relaxation rate (1/*T*_1_) and Gd^3+^ concentrations in GO-AuNS-DOTA-Gd aqueous solutions; (h) representative vertical PET images of a mouse with tumor after ^18^F-FDG injection.

PAI and MRI were performed to investigate targeting ability of GO-AuNS-DOTA-Gd in tumor-bearing mice. Figures 4(a) and 4(c) depict drug concentration within tumor as a function of time before and after tail-vein injection of probe and quantitative analysis of the corresponding imaging using PAI. The signals in cross-sectional images of tumor region of interest (tumor ROI) started to accumulate from 2 h and reached a peak at 5 h with a nearly 2.4-fold enhancement compared with that of preinjection until 24 h in close proximity to that of preinjection.

Figures 4(b) and 4(d) depict MRI imaging and quantitative analysis of average gray of tumor ROI using MRI, respectively. The gray value in the coronal plane images of tumor ROI on 5th h obviously exceeded the value on the other three time points. The relaxivity of *T*_1_ was measured to be 1.044*e* − 5 *μ*M^−1^·ms^−1^ (Figures 4(f) and 4(g)). In contrast with ^18^F-FDG PET image (Figure 4(h)), the MRI image after probe injection allowed us to distinguish tumor margin from the normal tissue. To quantitatively analyze the probe's ability of tumors targeting and enhancement, the tumor was segmented and visualized using 3D slicer (Figure 4(e)). The calculated tumor volume and maximal radium on 5 h exceeded that of preinjection and on 2 h and then the value declined slowly over 5 h (Table 1). Gray contrast ratio of tumor versus peripheral tissue on 5 h was higher than that on the other three time points. The results indicated that probe concentration in the tumor on 5 h reached a peak and presented the best ability to distinguish the tumor from normal tissues.

### 3.4. In Vivo Antitumor Efficacy Evaluation Based on Image Data Analysis

**Figure 5:**
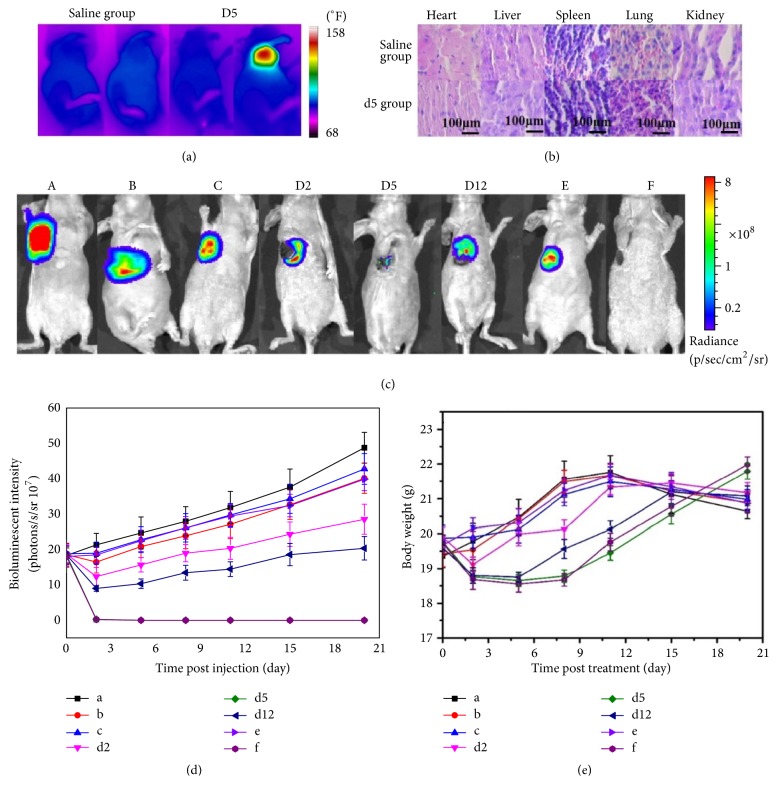
(a) Thermal image of mice before and after irradiation for the saline group and (D5) group; (b) sections of the heart, liver, spleen, lung, and kidney of mice stained with H&E after saline and GO-AuNS treatment; (c) bioluminescence images of the HCC-LM3-fLuc tumor-bearing nude mice that received (A) saline through tail vein without laser irradiation, (B) saline through tail vein with laser irradiation, (C) GO-AuNS through tail vein without laser irradiation, (D2) laser irradiation on 2nd h postinjection of GO-AuNS through tail vein, (D5) laser irradiation on 5th h postinjection of GO-AuNS through tail vein, (D12) laser irradiation on 12th h postinjection of GO-AuNS through tail vein, (E) GO-AuNS via intratumor injection without laser irradiation, and (F) laser irradiation on 2nd h postinjection of GO-AuNS via intratumor injection; (d) the quantified bioluminescence intensity of tumors from the eight groups on day 5 posttreatment; (e) the average weight of mice from the eight groups over time (*n* = 5 per group).

The temperature of tumor on GO-AuNS-DOTA-Gd treated mice ((D2), (D5), (D12), and (F)) reached more than 140°F after irradiation ((D2): 140°F, (D5): 148°F, (D12): 145°F, and (F): 149°F), while GO-AuNS-DOTA-Gd tail-injected and intratumor injected without irradiation and all saline treated mice with and without irradiation showed no temperature increment (Figure 5(a) only shows saline control group and (D5) group). As shown in Figure 5(c), the bioluminescence intensity of the mice of (D5) and (F) groups was zero on day 5 after treatment, leaving the original tumor sites as black scars. The quantitative analysis of the eight groups demonstrated that treatment with laser irradiation on tumor at 5th h after probe injection via tail vein achieved the same antitumor effect as laser irradiation treatment on tumor after intratumor injection (Figure 5(d)). Compared with the current photothermal methods (for (D12) group, if the therapy was initiated on 12th h postinjection (7-8)), this synergistic method based on multimodality molecular imaging (for (D5) group) could improve treatment efficacy by 36.36% on 2nd day evaluated by BLI for subcutaneous tumor. Tumor growth for groups (D2) and (D12) was inhibited after treatment until day 4 and then tumor grew slowly close to that of the other groups.

**Figure 6:**
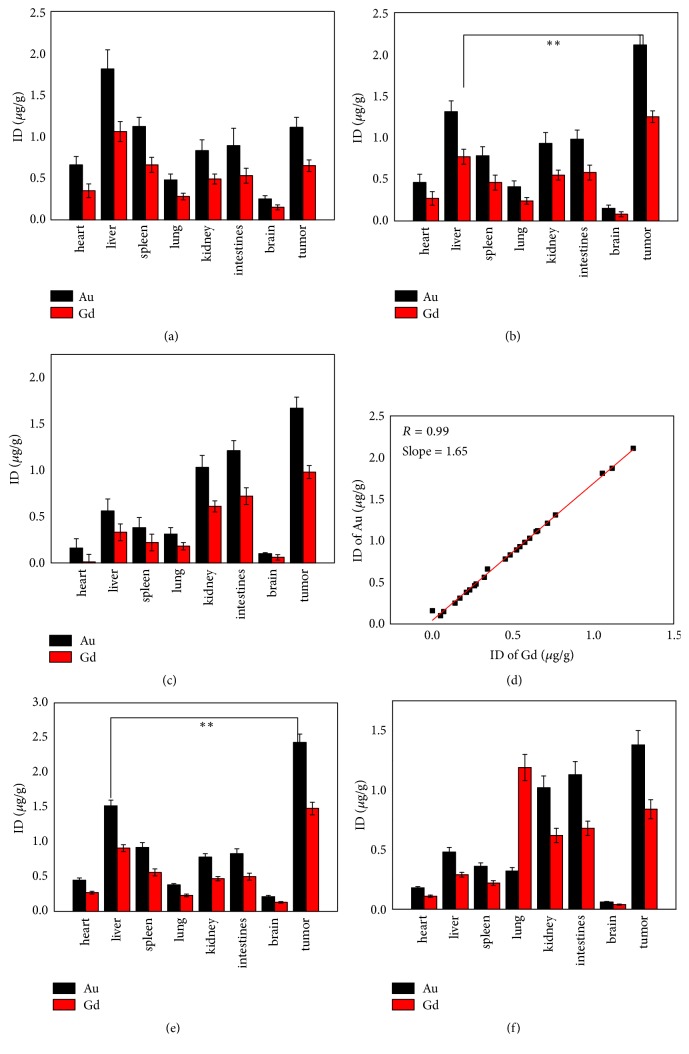
Quantitative tissue biodistribution of Au and Gd in HCC-LM3-fLuc subcutaneous tumor-bearing mice after intravenous injection: 2 h (a), 5 h (b), and 12 h (c). Data was presented as mean ± SD (*n* = 5), ^*∗∗*^*p* < 0.01, significant difference compared with the other tissue distribution of Au and Gd; (d) the correlation between concentrations of Au and Gd. (e) and (f) Quantitative tissue biodistribution of Au and Gd in HCC-LM3-fLuc orthotopic tumor-bearing mice after intravenous injection: 1 h (e) and 12 h (f).

To evaluate the toxicity of the probe, body weight of mice was monitored throughout the antitumor study. None of mouse weight in the eight groups declined more than 20% of its original weight (Figure 5(e)). H&E staining of the heart, liver, spleen, lung, and kidney of the mice showed no distinct damage for all drug treatment groups (Figure 5(b), only showing (D5) group and saline control group). ICP results demonstrated that the probe concentration in the tumor reaches to the summit on 5th h and probe concentration in liver and spleen gradually declined postinjection (shown in Figures 6(a), 6(b), and 6(c)). The concentration of Au NPs is linearly dependent on the concentration of Gd with 0.99 correlation coefficient and 1.65 slope which is nearly consistent with mass ratio of Au NPs and Gd determined by ICP (in Result1, Au/Gd = 1.31 : 0.77).

### 3.5. Multimodality Molecular Imaging-Guided Orthotopic Tumor Treatment

**Figure 7:**
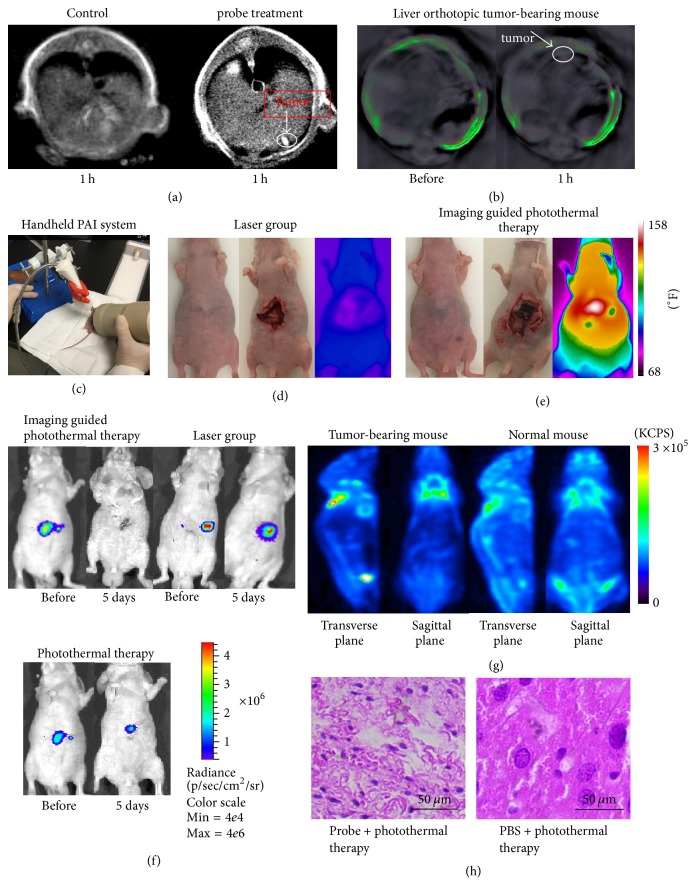
(a) MRI image of GO-AuNS-DOTA-Gd on the coronal plane in the tumor regions on 1st h postinjection; (b) photoacoustic signal distribution of GO-AuNS-DOTA-Gd in the tumor regions on 1st h postinjection; (c) handheld photoacoustic imaging-guided photothermal therapy; (d) and (e) are mice image before treatment, treatment, and after irradiation of PBS group and probe group with handheld PAI guidance; (f) bioluminescence image of mice from probe with PAI guidance, PBS group, and probe without PAI guidance; (g) ^18^F-FDG PET image of liver orthotopic tumor-bearing mouse and normal mouse; (h) H&E staining of tumors posttreatment from control group and probe group.

Figures 7(a) and 7(b) demonstrated that GO-AuNS-DOTA-Gd can target liver orthotopic tumor and reach to the top concentration on 1st h postinjection via tail vein.  Figure 7(c) depicted the treatment process using handheld photoacoustic imaging. During treatment, the temperature of the liver tumor on GO-AuNS-DOTA-Gd treated mice and reached more than 150°F after irradiation, while the temperature of liver tumor on PBS treated mice did not show obvious temperature increment (Figures 7(d) and 7(e)). Compared with PBS group, tumor of mice for probe group was inhibited completely on day 5th after treatment under handheld PAI guidance (antitumor efficacy is 100%). Without handheld PAI guidance, residual tumors were found on six of seven mice after probe injection and photothermal therapy (antitumor efficacy is 14.29%) (Figure 7(f)). ICP results based on the orthotopic mouse demonstrated that the probe can be concentrated in the tumor site at 1st h (shown in Figures 6(e) and 6(f)). All experiment results demonstrated that this synergistic method could effectively reduce tumor residual and satellite metastases by 85.71% compared with the photothermal method without handheld PAI guidance (100%  − 14.29% = 85.71%). On the other hand, ^18^F-FDG PET image cannot distinguish the tumor from the liver so it cannot be used for guidance in the treatment (Figure 7(g)). The H&E staining result demonstrated that the tumor cells were damaged by the photothermal therapy after probe injection, while the tumor cells were kept in good condition for PBS group.

## 4. Discussion

Graphene has been used for photothermal therapy and has shown promise in many* in vitro* and* in vivo* studies [18, 19]. However, photothermal therapy based on graphene and its related detailed imaging analysis, including tumor volume and gray contrast ratio, have been rarely reported [20]. In this study, GO-AuNS-DOTA-Gd was prepared and its physicochemical properties indicated that the GO-AuNS-DOTA could act as an efficient material for photothermal therapy and be used for precisely delineating the tumor margin from normal tissues. GO is believed to have a role in promoting Au nanostar photothermal transduction efficacy in certain studies which deserved further study [21].

Determining tumor boundary is important and can influence the tumor surgery and treatment [22]. In contrast with ^18^F-FDG PET imaging, MRI imaging based on GO-AuNS-DOTA-Gd can accurately distinguish the tumor from normal tissues. Furthermore, MRI and PAI analysis demonstrated that the concentration of GO-AuNS-DOTA-Gd reached its peak in tumor regions at 5th h for subcutaneous tumor and 1st h for orthotopic tumor after tail-vein injection, which was determined as the optimal implementing time of photothermal therapy. For orthotopic tumor, handheld PAI guided photothermal therapy can effectively reduce the tumor residual and ensured the killing of tumor cells.

Molecular imaging has been widely used in tumor research [23–25]. However, reports on multimodality molecular imaging-guided tumor margin delineation and tumor treatment with deep tumor feature analysis are scarce. Imaging analysis has gradually showed its importance not only for subcutaneous tumor but also for orthotopic tumor and some groups developed image processing methods to resolve problems encountered in tumor surgery and cancer research. However, there are still some technological difficulties to be resolve, including quick acquisition of massive data and image segmentation accuracy [24, 25]. In the future, with the development of multidiscipline research, better solutions will be developed to solve biomedical problems using more mature imaging technologies.

## 5. Conclusions

In this study, GO-AuNS-DOTA-Gd was prepared and its physicochemical properties indicated that the probe could act as an efficient photosensitizer for photothermal therapy and be used for precisely delineating the tumor margin from normal tissues based on multimodality molecular imaging. This multimodality imaging strategy is a promising technology for clinical use, especially for the orthotopic tumor, which deserves further development for application in other tumor surgery and treatment.

## Figures and Tables

**Table 1:** Maximal radium of tumor, tumor volume, and gray contrast ratio of tumor versus other tissue around the tumor based on MRI.

	Pre	2 h	5 h	12 h
Tumor volume (mm^3^)	249.32	249.53	289.39	271.70
Maximal diameter (mm)	6.824	7.086	7.345	7.331
Gray contrast ratio	1.3520	1.3573	1.8533	1.5343

*Note*. One voxel is 0.1 × 0.065625 × 1 mm^3^ according to the MRI image.
